# Real-world use of temsirolimus in Japanese patients with unresectable or metastatic renal cell carcinoma: recent consideration based on the results of a post-marketing, all-case surveillance study

**DOI:** 10.1093/jjco/hyaa062

**Published:** 2020-05-27

**Authors:** Shigeru Sugiyama, Kazuo Sato, Yoshiyuki Shibasaki, Yutaka Endo, Taku Uryu, Yasuharu Toyoshima, Mototsugu Oya, Naoto Miyanaga, Nagahiro Saijo, Akihiko Gemma, Hideyuki Akaza

**Affiliations:** 1 Pfizer Japan Inc., Tokyo, Japan; 2 Pfizer R&D G.K., Tokyo, Japan; 3 Department of Urology, Keio University Hospital, Tokyo, Japan; 4 Department of Urology, Mito Saiseikai General Hospital, Ibaraki, Japan; 5 Japanese Society of Medical Oncology, Tokyo, Japan; 6 Department of Pulmonary Medicine and Oncology, Graduate School of Medicine, Nippon Medical School, Tokyo, Japan; 7 Interfaculty Initiative in Information Studies, Graduate School of Interdisciplinary Information Studies, The University of Tokyo, Tokyo, Japan

**Keywords:** carcinoma renal cell, Japan, safety, product surveillance postmarketing, temsirolimus

## Abstract

**Objective:**

A prospective, observational, post-marketing surveillance was conducted to assess the safety and effectiveness of temsirolimus in patients with renal cell carcinoma in Japan.

**Methods:**

Patients prescribed temsirolimus for advanced renal cell carcinoma were registered and received temsirolimus (25 mg weekly, intravenous infusion for 30–60 minutes) in routine clinical settings (observation period: 96 weeks).

**Results:**

Among 1001 patients included in the safety analysis data set (median age, 65.0 years; men, 74.8%; Eastern Cooperative Oncology Group performance status 0 or 1, 69.6%), 778 (77.7%) reported adverse drug reactions. The most common (≥10%) all-grade adverse drug reactions were stomatitis (26.7%), interstitial lung disease (17.3%) and platelet count decreased (11.1%). The incidence rate of grade ≥3 interstitial lung disease was 4.5%. The onset of interstitial lung disease was more frequent after 4–8 weeks of treatment or in patients with lower Eastern Cooperative Oncology Group performance status (21.6% for score 0 vs 8.3% for score 4, *P* < 0.001). Among 654 patients in the effectiveness analysis data set, the response and clinical benefit rates were 6.7% (95% confidence interval 4.9–8.9) and 53.2% (95% confidence interval 49.3–57.1), respectively. The median progression-free survival was 18.3 weeks (95% confidence interval 16.9–21.1).

**Conclusions:**

The safety and effectiveness profile of temsirolimus observed in this study was similar to that observed in the multinational phase 3 study. The results are generalizable to the real-world scenario at the time of this research, and safety and effectiveness of temsirolimus as a subsequent anticancer therapy for renal cell carcinoma warrants further investigation. (ClinicalTrials.gov identifier NCT01210482, NCT01420601).

## Teaser

In this Japanese post-marketing surveillance study, the safety and effectiveness profile of temsirolimus was similar to that in the multinational phase 3 study. No new safety signals were identified.

## Introduction

Kidney cancer has an estimated worldwide annual incidence of ~270 000 cases, making it one of the common malignancies (1). In Japan, the age-standardized incidence of kidney cancer is increasing annually (5.2 per 100 000 persons in 2005, 6.6 per 100 000 persons in 2010 and 8.0 per 100 000 persons in 2015), and its age-standardized mortality rate in 2017 was 1.8 per 100 000 persons (2.8 per 100 000 persons in men and 1.0 per 100 000 persons in women) (2). Approximately 90% of kidney cancers are classified as renal cell carcinoma (RCC) (1). Owing to the technological advances in oncology in the past 10 years, high price tags typically associated with new medical technologies and the emergence of new oncologic technologies for the treatment of RCC, the economic burden of RCC has become considerably higher than before (3). Moreover, 25–30% of patients with RCC present with metastatic disease at the time of diagnosis (4), highlighting the need for systemic therapies as the mainstay of treatment for RCC.

The strategy for the systemic treatment of RCC has changed dramatically in the past decade from cytokine-based therapy to molecular-targeted therapy. Currently, in Japan, tyrosine kinase inhibitors (sorafenib, sunitinib, pazopanib and axitinib), mammalian target of rapamycin (mTOR) inhibitors (everolimus and temsirolimus) and immune checkpoint inhibitors (nivolumab and ipilimumab) are available for the treatment of unresectable or metastatic RCC (5,6).

Temsirolimus is recommended as the first-line treatment for RCC in the guidelines of the Japanese Urological Association (high-risk, clear-cell, advanced RCC and non-clear cell, advanced RCC) (7), the United States National Comprehensive Cancer Network (poor-risk, relapsed or medically unresectable, clear-cell, stage IV RCC) (8) and the European Society for Medical Oncology (poor-risk, clear-cell RCC) (9). The efficacy and safety of temsirolimus in RCC was reported in a nonrandomized, phase 2 study conducted in East Asian patients with advanced RCC (10) and a multinational, randomized, phase 3 study involving 626 patients with previously untreated, poor-prognosis metastatic RCC (11). In Japan, temsirolimus was well tolerated in routine clinical settings as demonstrated in a single-center, retrospective study involving 55 patients with metastatic RCC (12) and in another study involving 10 patients with metastatic RCC undergoing hemodialysis (13). However, evidence from large-scale studies on the safety and effectiveness of temsirolimus in Japanese patients with RCC, including the onset of drug-induced interstitial lung disease (ILD) (14,15), remains limited.

Temsirolimus was approved in July 2010 in Japan for the treatment of advanced RCC (6). To assess the safety and effectiveness of temsirolimus in real-world clinical settings in Japan, a prospective, observational, post-marketing surveillance (PMS) study was conducted as part of a mandatory, post-approval regulatory requirement. An all-case surveillance methodology (16) was adopted to accumulate data on the real-world safety and effectiveness of temsirolimus as early as possible and to ensure that necessary measures are taken for the proper use of temsirolimus in patients with RCC in Japan (ClinicalTrials.gov identifier NCT01210482 and NCT01420601).

## Patients and methods

### Study design

An open-label, single-arm design was employed in this study, which consisted of a 24-week, all-case surveillance period and an optional, long-term treatment period. The observation period was 96 weeks after starting temsirolimus treatment (Supplementary Fig. 1). In patients whose treatment was discontinued before week 96, the observation period continued for 28 days after discontinuation. Of note, the post-approval regulatory requirement for conducting the all-case surveillance for temsirolimus in patients with RCC was lifted in December 2016, as the Japanese regulatory authority confirmed that sufficient data have been accumulated on the real-world safety and effectiveness of temsirolimus, and necessary measures have been taken for the proper use of the drug in this patient population.

This investigation was performed in accordance with the ministerial ordinance on Good Post-marketing Study Practice in Japan (Ordinance No. 171; 20 December 2004).

### Patients

Patients prescribed temsirolimus (25 mg infusion, TORISEL®, Pfizer Japan Inc.) for its approved indication (unresectable or metastatic RCC) were registered using a central registration system at sites where physicians had sufficient knowledge of and experience with the pharmacotherapy of renal cancer. Among the registered patients, those who continued treatment with temsirolimus for more than 24 weeks were transitioned to the long-term treatment period and observed for up to 96 weeks.

### Treatment

Patients received temsirolimus 25 mg weekly by intravenous infusion for 30–60 minutes. The dosage was reduced appropriately depending on the patients’ condition. When mild clinical symptoms (e.g. dyspnoea and cough) suggestive of ILD developed, temsirolimus was discontinued until the symptoms were relieved. Administration of temsirolimus was permanently discontinued in patients who had severe clinical symptoms (e.g. dyspnoea and cough) requiring oxygen therapy, when symptoms exacerbated with reduced lung diffusing capacity or when any change in clinical or imaging findings was observed in patients with underlying lung diseases. When any grade ≥3 adverse drug reaction [ADR; i.e. adverse event (AE) having a potential causal relationship with temsirolimus] other than ILD developed, administration of temsirolimus was discontinued until the ADR was resolved. Physicians and pharmacists were informed about the safety and efficacy of temsirolimus through package inserts, precautions for use, interview forms, summary of general information and proper use guides.

### Endpoints

The safety endpoint was the incidence of ADRs. Furthermore, in consideration of common AEs associated with temsirolimus and mTOR inhibitors (10,11,14,15), ILD, dyspnoea, diabetes mellitus/hyperglycaemia, hypersensitivity reaction, diarrhoea, hypophosphataemia, hypokalaemia, hypercholesterolaemia/hyperlipidaemia, infections, intracerebral haemorrhage, abnormal wound healing, mucositis-related ADRs, skin disorder, acute renal failure, gastrointestinal perforation and history of infection (hepatitis B, tuberculosis or herpes zoster) were monitored as major investigation items. Effectiveness endpoints were response rate [percentage of patients with complete response (CR) or partial response (PR) as best overall response] and clinical benefit rate [CBR; percentage of patients with CR, PR or stable disease (SD) maintained for ≥24 weeks as best overall response]. Tumor evaluation [CR, PR, progressive disease (PD), SD or not evaluable] was performed as per the Response Evaluation Criteria in Solid Tumors (RECIST) guideline version 1.1 (17,18). Progression-free survival (PFS), time to onset of ILD and background factors that may affect the incidence of ILD were examined as exploratory endpoints. PFS was defined as the time from the initiation of treatment to PD or death from any cause. If PD or death was not observed, patients were censored at their last date of tumor response evaluation. Patients without any record on tumor response evaluation were censored at the date of treatment initiation with temsirolimus.

### Assessments

Patient information, including demographics and baseline characteristics, dosage and AEs, was recorded on case report forms (CRFs). Patient background, medical history, treatment history and registration information, including scheduled date of initiation of treatment and availability of pretreatment chest computed tomography (CT), were recorded at baseline. Thereafter, safety was assessed using chest CT, chest X-rays and clinical laboratory tests, and by monitoring of AEs and ADRs during the observation period.

AEs and ADRs were monitored for 96 weeks after starting temsirolimus treatment or for 28 days after early discontinuation. Tumor progression was not recorded as an AE except the event leading to death during the observation period. When a patient started a new treatment regimen because of an AE, AE monitoring in this study was terminated. Tumor evaluation was performed at weeks 8, 16 and 24. Tumor evaluation in patients discontinuing temsirolimus early was performed either at discontinuation or at the last visit before discontinuing (within 4 weeks before discontinuation).

### Statistical analysis

To detect ≥1 case with the event at a probability of ≥95%, assuming a true AE incidence of 1.0%, the target sample size was set to 600 to collect data for 300 patients treated with temsirolimus for ≥12 weeks, assuming a completion rate of 50%. However, because of a lower-than-expected completion rate (~36%), the target sample size was raised to 1000 to secure at least 300 patients treated for ≥12 weeks.

The safety analysis data set comprised all patients who received ≥1 dose of temsirolimus. The effectiveness analysis data set comprised all patients in the safety analysis data set but excluded those receiving temsirolimus as an off-label use, those previously treated with temsirolimus and those who did not undergo effectiveness evaluations or did not provide effectiveness results. Risk classification was performed using the Memorial Sloan Kettering Cancer Center (MSKCC) model (19) and its modified 6-factor model (20). ADRs were coded using the Medical Dictionary for Regulatory Activities version 20.1 and graded per the Common Terminology Criteria for Adverse Events (CTCAE) version 4.0. The response rate and CBR were calculated from the tumor evaluation results rated as per the RECIST guideline. The 95% two-sided confidence interval (CI) was estimated by the Clopper–Pearson method. Fisher’s exact test and the Cochran–Armitage test were used to evaluate categorical and ordinal scale data, respectively, with a significance level of 5%. Median PFS was estimated by the Kaplan–Meier method.

## Results

### Patient disposition and baseline characteristics

This PMS study was conducted at 420 sites in Japan, which had a contract with the sponsor for the conduct of this study (study period: September 2010–March 2014). Among the 1050 registered patients, 29 were excluded because of no drug administration (27 patients) or duplicated registration (2 patients), and CRFs were not collected for 18 patients. Consequently, CRFs were collected from 1003 patients; however, 2 patients were excluded from the safety analysis data set (breach of contract and no drug administration, 1 patient each), resulting in 1001 patients. A total of 347 patients were excluded from the effectiveness analysis, resulting in 654 patients in the effectiveness analysis data set (Supplementary Fig. 2).

Among the 1001 patients included in the safety analysis, 749 (74.8%) were men, and the median age (range) was 65.0 (1–89) years. The Eastern Cooperative Oncology Group performance status (ECOG PS) was 0 or 1 in 697 (69.6%) patients. A total of 213 (21.3%) patients were rated as poor risk based on the MSKCC risk classification (Table 1).

**Table 1:** Patient demographics and baseline characteristics

Characteristics	Safety analysis data set (*N* = 1001)
Men	749 (74.8)
Age, years	
<45	90 (9.0)
≥45 to <55	101 (10.1)
≥55 to <65	301 (30.1)
≥65 to <75	309 (30.9)
≥75 to <85	176 (17.6)
≥85	8 (0.8)
Unknown	16 (1.6)
Mean ± standard deviation^*^	63.4 ± 12.56
Median (minimum, maximum)^*^	65.0 (1, 89)
ECOG PS	
0	370 (37.0)
1	327 (32.7)
2	178 (17.8)
3	100 (10.0)
4	24 (2.4)
Unknown	2 (0.2)
MSKCC risk	
Favorable	13 (1.3)
Intermediate	342 (34.2)
Poor	213 (21.3)
Unknown	433 (43.3)
Dosing period, weeks	
Mean ± standard deviation	16.5 ± 18.4
Median (minimum, maximum)	11.0 (1, 100)

^*^ *n* = 985.

Data are represented as *n* (%) unless otherwise specified.

ECOG PS, Eastern Cooperative Oncology Group performance status; MSKCC, Memorial Sloan Kettering Cancer Center.

Patients received temsirolimus for a median (range) of 11.0 (1–100) weeks (Table 1). Among the 1001 patients included in the safety analysis, 914 (91.3%) discontinued treatment with temsirolimus, with the most common reasons being insufficient effectiveness (423 patients), AEs (301 patients) and death (125 patients).

### Safety

A total of 2166 ADRs were reported in 778 (77.7%) patients. The most common (≥10%) all-grade ADRs were stomatitis (267 [26.7%] patients), ILD (173 [17.3%] patients) and platelet count decreased (111 [11.1%] patients) (Table 2). Among the major investigation items, the most common (≥15%) all-grade ADRs were mucositis-related ADRs (279 [27.9%] patients), skin disorder (209 [20.9%] patients) and ILD (174 [17.4%] patients, including 1 patient with pneumonitis), and the most common grade ≥3 ADRs were infections (68 [6.8%] patients), ILD (45 [4.5%] patients) and diabetes/hyperglycaemia (40 [4.0%] patients) (Table 3). Among 28 patients with a history of infection with hepatitis B (8 patients), tuberculosis (14 patients) or herpes zoster (6 patients), ADRs were reported in 26 (92.9%) patients.

**Table 2:** ADRs reported by ≥5% of patients

Events	Safety analysis data set (*N* = 1001)
	All grade
Number of patients with ADRs, *n* (%)	778 (77.7)
Number of ADRs, *n*	2166
ADRs (all grade, ≥5% of patients), *n* (%)	
Stomatitis	267 (26.7)
ILD	173 (17.3)
Platelet count decreased	111 (11.1)
Hyperglycaemia	98 (9.8)
Rash	74 (7.4)
Anaemia	63 (6.3)
Hyperlipidaemia	61 (6.1)
Hypercholesterolaemia	55 (5.5)

ADR, adverse drug reaction; ILD, interstitial lung disease.

**Table 3:** Safety profile for major investigation items

Events, *n* (%)	Safety analysis data set (*N* = 1001)		
	All ADRs	Serious ADRs	Grade ≥ 3 ADRs
Number of patients with ADRs	653 (65.2)	280 (28.0)	226 (22.6)
ILD^*^	174 (17.4)	172 (17.2)	45 (4.5)
Diverse events suspected of ILD	3 (0.3)	2 (0.2)	−
Dyspnoea	35 (3.5)	21 (2.1)	19 (1.9)
Diabetes/hyperglycaemia	131 (13.1)	17 (1.7)	40 (4.0)
Hypersensitivity	11 (1.1)	7 (0.7)	5 (0.5)
Diarrhoea	43 (4.3)	4 (0.4)	6 (0.6)
Hypophosphataemia	39 (3.9)	2 (0.2)	16 (1.6)
Hypokalaemia	14 (1.4)	1 (0.1)	2 (0.2)
Hypercholesterolaemia/hyperlipidaemia	140 (14.0)	3 (0.3)	19 (1.9)
Infections	141 (14.1)	65 (6.5)	68 (6.8)
Intracerebral haemorrhage	1 (0.1)	1 (0.1)	−
Abnormal wound healing	1 (0.1)	−	−
Mucositis-related AEs	279 (27.9)	17 (1.7)	38 (3.8)
Skin disorder	209 (20.9)	6 (0.6)	18 (1.8)
Acute renal failure	17 (1.7)	13 (1.3)	11 (1.1)
Gastrointestinal perforation	1 (0.1)	1 (0.1)	1 (0.1)

^*^Including 1 patient with pneumonitis.

ADR, adverse drug reaction; AE, adverse event; ILD, interstitial lung disease.

As the onset of major investigation items was recorded independently from the onset of ADRs, the number of patients reporting major investigation items may not be the same as that with ADRs.

A total of 496 serious ADRs were reported in 352 (35.2%) patients; the most common (≥2%) events were ILD (172 [17.2%] patients) and dyspnoea (20 [2.0%] patients). A total of 246 unexpected ADRs were reported in 191 (19.1%) patients. The most common (≥1%) events were palmar-plantar erythrodysaesthesia syndrome (16 [1.6%] patients), hyperkalaemia (13 [1.3%] patients) and dyspnoea, constipation and C-reactive protein increased (11 [1.1%] patients each) (Supplementary Table 1). A potentially drug-related death (CTCAE grade 5) was reported in 32 (3.2%) patients, the most commonly reported preferred terms being ILD (9 [0.9%] patients) followed by *Pneumocystis jirovecii* pneumonia and death (4 [0.4%] patients each) (Supplementary Table 2).

**Figure 1.**
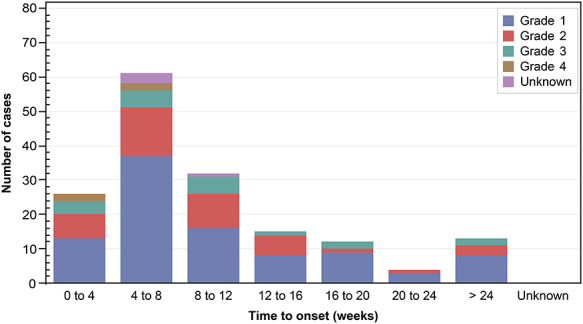
ILD occurrence summarized by time to onset (*n* = 174, no data are available for 11 cases, safety analysis data set). ILD, interstitial lung disease.

Among 174 patients who reported ILD, 82 (47.1%) had a grade 1 event, of whom 26 continued temsirolimus. Among 46 (26.4%) patients with grade 2 ILD, 1 patient continued temsirolimus. Grades 3, 4 and 5 ILD were reported in 30 (17.2%), 6 (3.4%) and 9 (5.2%) patients, respectively, for whom temsirolimus dose was reduced, withdrawn or discontinued (Supplementary Table 3). The onset of ILD was most frequently observed at 4–8 weeks after starting temsirolimus (Fig. 1). When stratified by patient characteristics, the proportion of patients who experienced ILD was significantly higher among those with advanced age (20.3% for age ≥ 65 years vs 13.7% for age 15 to <65 years, *P* = 0.005), comorbidities (19.3 vs 14.1%, *P* = 0.038), comorbid lung disease (36.2 vs 16.5%, *P* = 0.001), comorbid ILD (53.3 vs 16.9%, *P* = 0.002), renal impairment (21.3 vs 15.4%, *P* = 0.022), lower ECOG PS (21.6% for score 0 vs 8.3% for score 4, *P* < 0.001) or longer disease duration (24.9% for ≥49 months vs 13.6% for ≤12 months, *P* < 0.001) (Table 4).

**Table 4:** Frequency of ILD stratified by baseline characteristics (safety analysis data set)

Variables	*N*	Patients with ILD onset, *n* (%)	*P* value
Age			
<15 years	3	0	0.016^*^
≥ 15 to <65 years	489	67 (13.7)	0.005^†^
≥65 years	493	100 (20.3)	
Unknown	16	7 (43.8)	
Comorbidities			
No	362	51 (14.1)	0.038^‡^
Yes	638	123 (19.3)	
Unknown	1	0	
Comorbid lung disease			
No	953	157 (16.5)	0.001^‡^
Yes	47	17 (36.2)	
Unknown	1	0	
Comorbid ILD			
No	985	166 (16.9)	0.002^‡^
Yes	15	8 (53.3)	
Unknown	1	0	
Renal impairment			
No	656	101 (15.4)	0.022^‡^
Yes	343	73 (21.3)	
Unknown	2	0	
ECOG PS			
0	370	80 (21.6)	0.003^*^
1	327	62 (19.0)	<0.001^†^
2	178	18 (10.1)	
3	100	12 (12.0)	
4	24	2 (8.3)	
Unknown	2	0	
Disease stage at diagnosis			
0	16	4 (25.0)	0.004^*^
1	86	18 (20.9)	0.003^†^
2	68	22 (32.4)	
3	142	29 (20.4)	
4	683	101 (14.8)	
Unknown	6	0	
Disease duration			
≤12 months	479	65 (13.6)	0.002^*^
13 to ≤24 months	152	29 (19.1)	<0.001^†^
25 to ≤48 months	161	37 (23.0)	
≥49 months	165	41 (24.9)	
Unknown	44	2 (4.6)	

^*^Fisher’s exact test (Monte Carlo method).

^†^Cochran–Armitage test (Monte Carlo method).

^‡^Fisher’s exact test.

ECOG PS, Eastern Cooperative Oncology Group performance status; ILD, interstitial lung disease.

Only statistically significant variables are presented.

### Effectiveness

**Figure 2.**
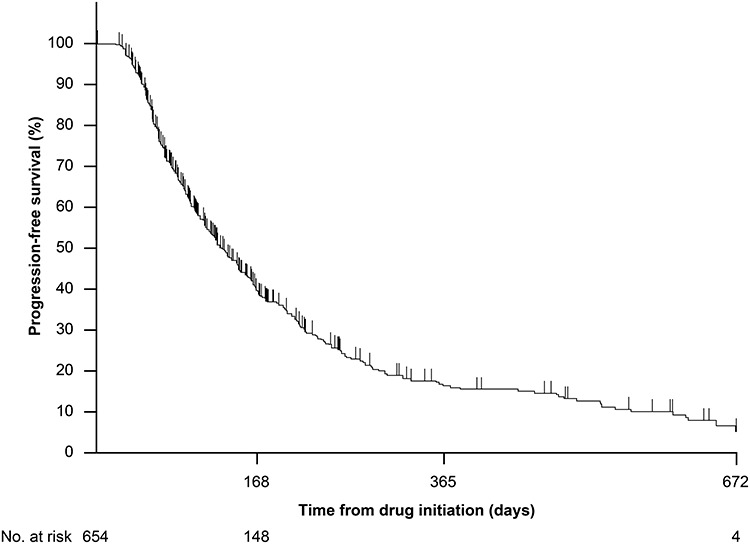
Progression-free survival (effectiveness analysis data set).

Among 654 patients in the effectiveness analysis data set, the response rate and CBR were 6.7% (95% CI 4.9–8.9) and 53.2% (95% CI 49.3–57.1), respectively (Table 5). The PFS rate at 24 weeks and 96 weeks of treatment was 39.2% (95% CI 34.8–43.5) and 5.0% (95% CI 2.2–9.7), respectively. The median PFS was 18.3 weeks (95% CI 16.9–21.1) (Fig. 2).

**Table 5:** Best overall response, response rate and CBR

Variables	Effectiveness analysis data set (*N* = 654)
Best overall response, *n* (%)	
CR	1 (0.2)
PR	43 (6.6)
SD	304 (46.5)
PD	243 (37.2)
Not evaluable	63 (9.6)
Response rate, *n* (%, 95% CI)^*^	44 (6.7, 4.9–8.9)
CBR, *n* (%, 95% CI)^†^	348 (53.2, 49.3–57.1)

^*^Patients with CR or PR.

^†^Patients with CR, PR or SD maintained for 24 weeks or longer.

CBR, clinical benefit rate; CI, confidence interval; CR, complete response; PD, progressive disease; PR, partial response; SD, stable disease.

## Discussion

More than 9 years have passed since the approval of temsirolimus in Japan in 2010 for the treatment of patients with RCC. However, evidence for its safety and effectiveness in routine clinical practice remains limited. Consequently, this all-case PMS study was conducted to assess the safety and effectiveness of temsirolimus in Japanese patients with advanced RCC.

The ADR profile observed in the current study was similar to that reported in previous studies, where the most common AEs reported in the multinational phase 3 study were asthenia (51%), rash (47%), anaemia (45%) and nausea (37%) (11), and the most common treatment-related AEs reported in the East Asian phase 2 study were rash (59%), stomatitis (57%), hypercholesterolaemia (43%), hypertriglyceridaemia (39%) and anorexia (37%) (10). However, the incidence rates of radiographically detected ILD in this study (all grade, 17.4%) and radiographically detected pneumonitis in the temsirolimus group of the phase 3 study (29%) were higher than those of investigator-identified pneumonitis in the temsirolimus group of the phase 3 study [2.4% (5/208 patients)] (14,21). This difference may be partly attributable to the difference in ILD diagnostic procedures between the current study and the previous phase 3 study. As the development of radiographically detected pneumonitis is commonly observed in patients with cancer treated with temsirolimus, physicians are advised to review chest CT images for radiographic signs of pneumonitis as part of close monitoring during treatment. Meanwhile, the response rate reported in the current study (6.7%) was slightly lower than that in the temsirolimus group of the previous phase 3 study (objective response rate 8.6%) (11). This response rate difference may be partly attributable to the difference in baseline patient characteristics between the current study and the previous phase 3 study, as illustrated by the baseline MSKCC risk classification: poor risk, 21% in this study vs 69% in the phase 3 study (11). Of note, in this surveillance, a considerably high proportion (43.3%) of patients was classified as unknown MSKCC risk. The MSKCC risk was assessed retrospectively based on the data collected during the surveillance (Karnofsky performance status, lactate dehydrogenase level, serum calcium level, hemoglobin level and time from diagnosis to systemic treatment), and patients were categorized as unknown MSKCC risk when data were unknown or missing for any of these 5 items. Therefore, further investigations were not feasible owing to a lack of data on the association of these characteristics with the effectiveness of temsirolimus.

In the current study, 17.4% (174/1001) of patients reported ILD as an ADR, with the majority [17.2% (172/1001)] reporting serious ADRs. Furthermore, the onset of ILD was more frequent within the first 4–8 weeks, which was consistent with the findings from a retrospective review of patients enrolled in the multinational phase 3 study of temsirolimus (estimated cumulative probability of temsirolimus-related pneumonitis, 21% [95% CI 15–29] at 8 weeks of treatment) (14). In addition, the results of our study showed that the onset of ILD was more frequent in patients with advanced age, comorbidities, renal impairment or longer disease duration, some of which were consistent with previous findings (22). Of note, 4 patients reported CTCAE grade 5 *P. jirovecii* pneumonia, for which a causal relationship with temsirolimus could not be ruled out. These findings are attributable to the difficulty of differentiating infections resulting from immunosuppressive treatment and mTOR inhibitor-induced lung diseases, highlighting the need to carefully monitor the onset of pneumonia in patients treated with temsirolimus.

In recent years, immune checkpoint inhibitors such as nivolumab plus ipilimumab (23), avelumab (24) and pembrolizumab (25) have emerged as a novel treatment option for previously untreated, advanced RCC owing to their demonstrated efficacy and safety. Accordingly, the value of temsirolimus as a first-line therapy for RCC may be decreasing. However, mTOR inhibitors, including temsirolimus, remain an attractive treatment option for RCC, particularly when immune checkpoint inhibitors are not available because of financial or regulatory constraints. In addition, the use of mTOR inhibitors following immune checkpoint inhibitors can be of benefit for RCC, given the demonstrated efficacy of temsirolimus in patients with poor-prognosis, metastatic RCC (11). Indeed, everolimus was among the three most commonly used subsequent anticancer therapies in the randomized clinical trials of avelumab plus axitinib (24) and pembrolizumab plus axitinib (25). Moreover, the intravenous administration route of temsirolimus is advantageous for patients with swallowing dysfunction, which may be caused by a number of factors, including anticancer therapies (26). Further research is warranted to investigate the efficacy of temsirolimus in subsequent line settings.

ILD is a safety concern commonly associated with mTOR inhibitors and immune checkpoint inhibitors (27). For example, nivolumab-related ILD has been reported in 7.2% (8/111 patients) of Japanese patients with non-small cell lung cancer involved in phase 2 studies (28). However, little is known about the impact of using mTOR inhibitors following immune checkpoint inhibitors and, in particular, whether their use generates any synergistic effects on the onset of ILD. Results from patient clinicopathological examination and experiments using a mouse model indicated an involvement of alveolar epithelial injury, via local and systemic lipid metabolic stress, in the pathogenesis of mTOR inhibitor-induced lung diseases (29), which may or may not differ from that induced by immune checkpoint inhibitors. Moreover, the pleiotropic effects of mTOR inhibition on multiple immune cell types (30) may unexpectedly be associated with the signaling pathways of programmed death-1 (PD-1) or programmed death ligand-1 (PD-L1). Indeed, a study using a tumor-bearing mouse model showed that blocking PD-L1 on tumor surface suppressed intracellular glycolysis by inhibition of mTOR activity, suggesting an association between the PD-L1 and mTOR signaling pathways (31). As the onset of ILD was more frequent in our patients with preserved physical status (i.e. lower ECOG PS), it could be argued that patients may experience ILD more frequently after their immune function is activated by immune checkpoint inhibition. However, owing to a lack of understanding of the etiology and mechanisms of ILD induced by immune checkpoint inhibitors (32), further research is required on the mode of action and potential risks for the use of mTOR inhibitors subsequent to immune checkpoint inhibitors. Consequently, for patients with RCC treated with temsirolimus following immune checkpoint inhibitors, it might be advisable to continue monitoring for ILD onset for 1–2 months following the initiation of temsirolimus treatment.

This study has some limitations. Firstly, the open-label, single-arm study design may have increased the risk of observational bias by the investigators. Secondly, tumor evaluation was based on investigators’ assessment, and no central assessment or independent review was employed. Thirdly, the possibility of underreporting of ADRs cannot be excluded (33), although this risk was mitigated by collecting the events in a solicited manner. Finally, findings of the current study may not be representative of the most up-to-date treatment experience of RCC in Japan as some time has elapsed since the study was conducted. However, as this study employed an all-case surveillance methodology, our results are highly generalizable to the real-world scenario in Japan at the time of this research and remain useful for the understanding of the safety and effectiveness of temsirolimus for the treatment of RCC in routine clinical settings.

In conclusion, the safety and effectiveness profile of temsirolimus observed in this PMS study was similar to that observed in the previous multinational phase 3 study. Overall, temsirolimus was well tolerated with no new safety signals.

## Supplementary Material

Supplementary_Figure_1_hyaa062

Supplementary_Figure_2_hyaa062

Supplementary_Tables_hyaa062
