# Supplementary figures and images for: Real-world use of temsirolimus in Japanese patients with unresectable or metastatic renal cell carcinoma: recent consideration based on the results of a post-marketing, all-case surveillance study
Source: Jpn J Clin Oncol. 2020 May 27;50(8):940–7. doi: 10.1093/jjco/hyaa062 (PMC7401718; doi:10.1093/jjco/hyaa062)

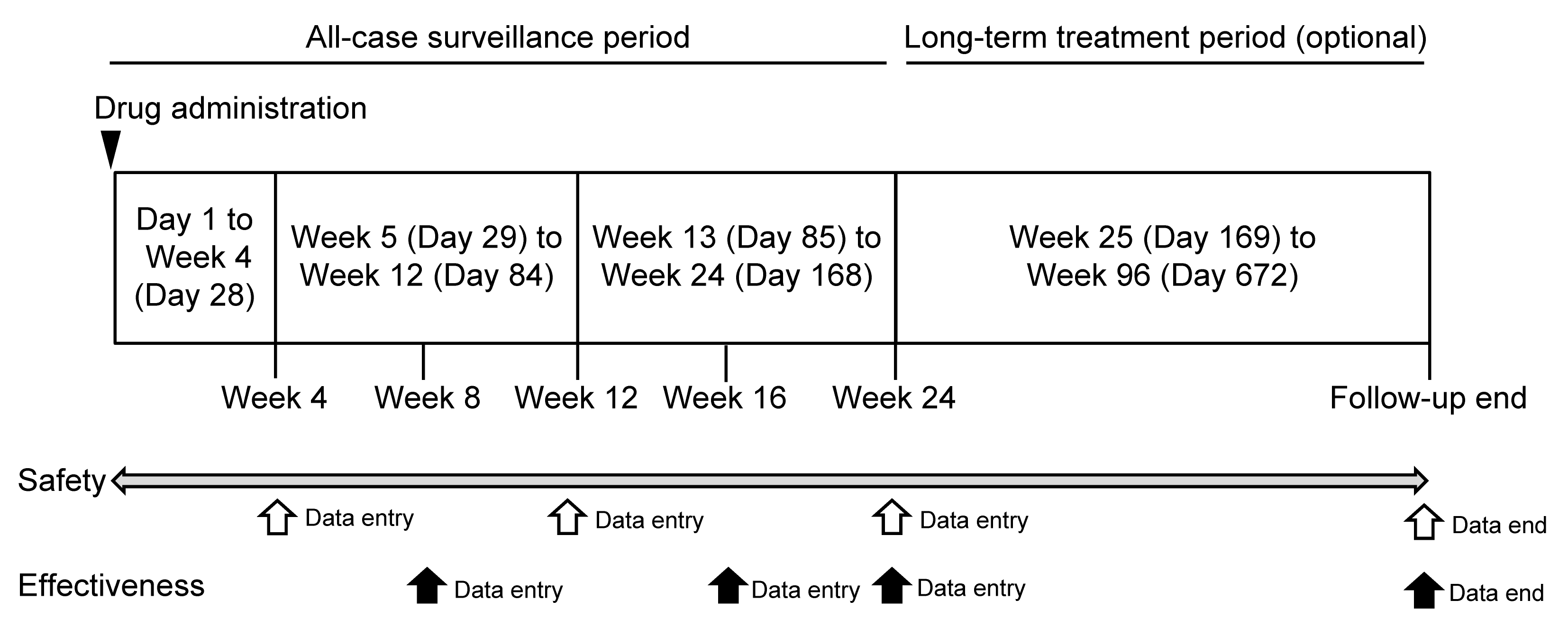

Supplement: Supplementary_Figure_1_hyaa062 [file supplementary_figure_1_hyaa062.png]

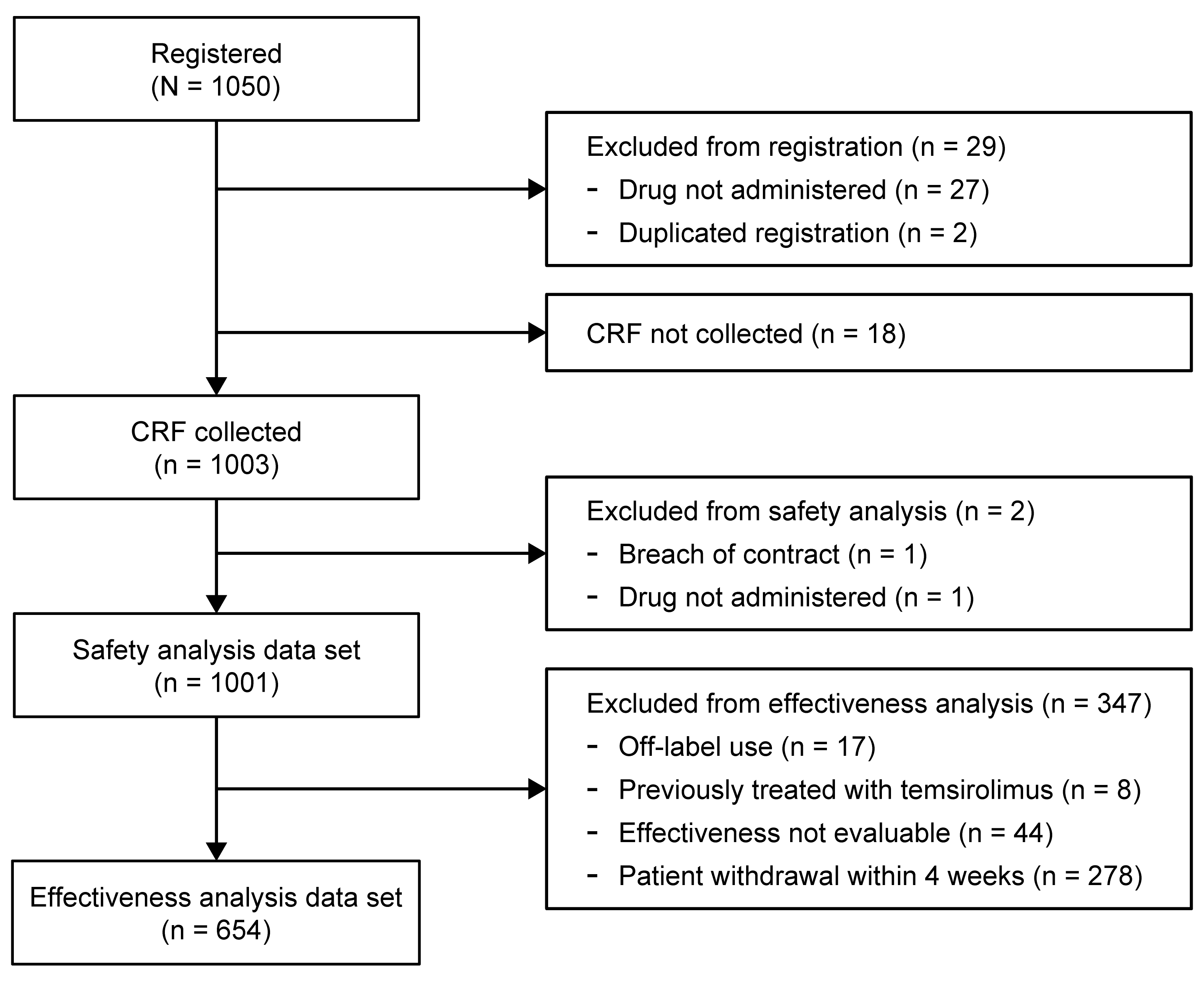

Supplement: Supplementary_Figure_2_hyaa062 [file supplementary_figure_2_hyaa062.png]
