# Supplementary material for: Real-world use of temsirolimus in Japanese patients with unresectable or metastatic renal cell carcinoma: recent consideration based on the results of a post-marketing, all-case surveillance study
Source: Jpn J Clin Oncol. 2020 May 27;50(8):940–7. doi: 10.1093/jjco/hyaa062 (PMC7401718; doi:10.1093/jjco/hyaa062)
Supplement: Supplementary_Tables_hyaa062 [file supplementary_tables_hyaa062.docx]

**Supplementary Table 1.** Serious and unexpected ADRs reported by ≥ 1% of patients

| Events | Safety analysis data set  (N = 1001) |
| --- | --- |
| Serious ADRs |  |
| Number of patients with serious ADRs, n (%) | 352 (35.2) |
| Number of serious ADRs, n | 496 |
| Serious ADRs (≥ 1% of patients), n (%) |  |
| ILD | 172 (17.2) |
| Dyspnoea | 20 (2.0) |
| Anaemia | 19 (1.9) |
| Pneumonia | 17 (1.7) |
| Stomatitis | 15 (1.5) |
| Pneumonia bacterial | 13 (1.3) |
| Hyperglycaemia | 12 (1.2) |
| *Pneumocystis jirovecii* pneumonia | 10 (1.0) |
| Renal failure acute | 10 (1.0) |
| Platelet count decreased | 10 (1.0) |
| Unexpected ADRs |  |
| Number of patients with unexpected ADRs, n (%) | 191 (19.1) |
| Number of unexpected ADRs, n | 246 |
| Unexpected ADRs (≥ 1% of patients), n (%) |  |
| Palmar-plantar erythrodysaesthesia syndrome | 16 (1.6) |
| Hyperkalaemia | 13 (1.3) |
| Dyspnoea | 11 (1.1) |
| Constipation | 11 (1.1) |
| C-reactive protein increased | 11 (1.1) |

ADR, adverse drug reaction; ILD, interstitial lung disease.

**Supplementary Table 2.** List of CTCAE grade 5 AEs

| Events | Safety analysis data set  (N = 1001) | |
| --- | --- | --- |
|  | **With causal relationship** | **Without causal relationship** |
| Number of patients with CTCAE grade 5 AEs, n (%) | 32 (3.2) | 153 (15.3) |
| Number of CTCAE grade 5 AEs, n | 39 | 171 |
| Infections and infestations | 12 (1.2) | 1 (0.1) |
| Pneumocystis jirovecii pneumonia | 4 (0.4) | - |
| Pseudomembranous colitis | 1 (0.1) | - |
| Pneumonia bacterial | 3 (0.3) | - |
| Sepsis | 2 (0.2) | - |
| Septic shock | 1 (0.1) | - |
| Pneumonia | 1 (0.1) | 1 (0.1) |
| Neoplasms benign, malignant and unspecified (incl cysts and polyps) | - | 4 (0.4) |
| Malignant ascites | - | 1 (0.1) |
| Lymphangiosis carcinomatosa | - | 2 (0.2) |
| Bile duct cancer | - | 1 (0.1) |
| Blood and lymphatic system disorders | 1 (0.1) | 4 (0.4) |
| Disseminated intravascular coagulation | 1 (0.1) | 3 (0.3) |
| Anaemia | - | 1 (0.1) |
| Metabolism and nutrition disorders | - | 2 (0.2) |
| Cachexia | - | 1 (0.1) |
| Hyponatraemia | - | 1 (0.1) |
| Nervous system disorders | - | 1 (0.1) |
| Hypoxic-ischaemic encephalopathy | - | 1 (0.1) |
| Cardiac disorders | 2 (0.2) | 4 (0.4) |
| Atrioventricular block complete | 1 (0.1) | - |
| Cardiac arrest | - | 1 (0.1) |
| Cardio-respiratory arrest | 1 (0.1) | 2 (0.2) |
| Cardiac failure | 1 (0.1) | 1 (0.1) |
| Respiratory, thoracic and mediastinal disorders | 12 (1.2) | 13 (1.3) |
| ILD | 9 (0.9) | - |
| Acute respiratory distress syndrome | - | 1 (0.1) |
| Dyspnoea | 3 (0.3) | 5 (0.5) |
| Respiratory disorder | - | 1 (0.1) |
| Respiratory failure | - | 4 (0.4) |
| Pneumonia aspiration | - | 2 (0.2) |
| Emphysema | 1 (0.1) | - |
| Pulmonary infarction | - | 1 (0.1) |
| Pulmonary embolism | 1 (0.1) | 1 (0.1) |
| Gastrointestinal disorders | 1 (0.1) | 1 (0.1) |
| Gastrointestinal haemorrhage | 1 (0.1) | - |
| Intestinal obstruction | - | 1 (0.1) |
| Renal and urinary disorders | 1 (0.1) | - |
| Acute kidney injury | 1 (0.1) | - |
| General disorders and administration site conditions | 7 (0.7) | 138 (13.8) |
| Death | 4 (0.4) | 1 (0.1) |
| Disease progression | 2 (0.2) | 137 (13.7) |
| Sudden death | 1 (0.1) | - |
| Investigations | - | 1 (0.1) |
| Nutritional condition abnormal | - | 1 (0.1) |

AE, adverse event; CTCAE, Common Terminology Criteria for Adverse Events; ILD, interstitial lung disease.

**Supplementary Table 3.** Onset of ILD stratified by CTCAE grade and temsirolimus dose change

| CTCAE grade | N | Temsirolimus dose after ILD onset, n | | | | | |
| --- | --- | --- | --- | --- | --- | --- | --- |
|  |  | **Continued** | **Reduced** | **Increased** | **Withdrawn** | **Discontinued** | **Unknown** |
| Grade 1 | 82 | 26 | 1 | 0 | 18 | 36 | 1 |
| Grade 2 | 46 | 1 | 0 | 0 | 9 | 36 | 0 |
| Grade 3 | 30 | 0 | 1 | 0 | 4 | 25 | 0 |
| Grade 4 | 6 | 0 | 0 | 0 | 0 | 6 | 0 |
| Grade 5 | 9 | 0 | 0 | 0 | 0 | 9 | 0 |
| Unknown | 1 | 0 | 0 | 0 | 0 | 1 | 0 |
| Total | 174 | 27 | 2 | 0 | 31 | 113 | 1 |

CTCAE, Common Terminology Criteria for Adverse Events; ILD, interstitial lung disease.
